# Divergence of exonic splicing elements after gene duplication and the impact on gene structures

**DOI:** 10.1186/gb-2009-10-11-r120

**Published:** 2009-11-02

**Authors:** Zhenguo Zhang, Li Zhou, Ping Wang, Yang Liu, Xianfeng Chen, Landian Hu, Xiangyin Kong

**Affiliations:** 1 The Key Laboratory of Stem Cell Biology, Institute of Health Sciences, Shanghai Institutes for Biological Sciences (SIBS), Chinese Academy of Sciences (CAS) and Shanghai Jiao Tong University School of Medicine (SJTUSM), 225 South Chong Qing Road, Shanghai 200025, PR China; 2 Graduate School of the Chinese Academy of Sciences, 19A Yuquan Road, Beijing 100049, PR China; 3 State Key Laboratory of Medical Genomics, Ruijin Hospital, Shanghai Jiaotong University, 197 Rui Jin Road II, Shanghai 200025, PR China

## Abstract

**Background:**

The origin of new genes and their contribution to functional novelty has been the subject of considerable interest. There has been much progress in understanding the mechanisms by which new genes originate. Here we examine a novel way that new gene structures could originate, namely through the evolution of new alternative splicing isoforms after gene duplication.

**Results:**

We studied the divergence of exonic splicing enhancers and silencers after gene duplication and the contributions of such divergence to the generation of new splicing isoforms. We found that exonic splicing enhancers and exonic splicing silencers diverge especially fast shortly after gene duplication. About 10% and 5% of paralogous exons undergo significantly asymmetric evolution of exonic splicing enhancers and silencers, respectively. When compared to pre-duplication ancestors, we found that there is a significant overall loss of exonic splicing enhancers and the magnitude increases with duplication age. Detailed examination reveals net gains and losses of exonic splicing enhancers and silencers in different copies and paralog clusters after gene duplication. Furthermore, we found that exonic splicing enhancer and silencer changes are mainly caused by synonymous mutations, though nonsynonymous changes also contribute. Finally, we found that exonic splicing enhancer and silencer divergence results in exon splicing state transitions (from constitutive to alternative or vice versa), and that the proportion of paralogous exon pairs with different splicing states also increases over time, consistent with previous predictions.

**Conclusions:**

Our results suggest that exonic splicing enhancer and silencer changes after gene duplication have important roles in alternative splicing divergence and that these changes contribute to the generation of new gene structures.

## Abstract 2

An analysis of human exonic splicing elements in duplicated genes reveals their important role in the generation of new gene structures.

## Background

There is an intimate link between the evolution of novel functions and the evolution of new genes [1-3]. Thus, it is important to understand the origin and evolution of new gene structures in order to understand the mechanisms responsible for the generation of new functions. Several mechanisms for the origin of new genes have been proposed, such as exon shuffling, gene duplication, and retroposition (reviewed in [4]). It is also possible that new gene structures are generated by the evolution of new alternative splicing forms after gene duplication. Consistent with this idea, 6 to 8% of profiled human-chimpanzee orthologous exons display significant splicing level differences in corresponding tissues. These genes affect diverse functions, including regulation of gene expression, signal transduction, cell death, immune defense, and susceptibility to diseases [5]. Previous studies have focused on the quantitative relationship between gene duplication and alternative splicing, and their findings support the quantitative divergence of alternative splicing events after gene duplication [6-9]. However, the details of this mechanism of alternative splicing evolution and its contributions to the evolution of new gene structures remain largely unknown.

Alternative splicing is a common and vital mechanism in higher eukaryotes that helps to increase transcriptional complexity [10,11]. By ligation of different exons or regions, alternative splicing is able to produce more than one transcript isoform from the same gene [10]. Alternative splicing requires a complex network and is regulated by many factors. In addition to sequences that comprise the branchpoint and the 3' and 5' splice sites, the cellular splicing machinery relies on additional information in the form of exonic splicing enhancer (ESE) and intronic splicing enhancer and exonic splicing silencer (ESS) and intronic splicing silencer sequences [10,12-14]. To carry out their functions, these elements are bound by different splicing factors, such as SR proteins [15] and heterogeneous nuclear ribonucleoproteins [10,16,17]. Several groups have made progress in the identification of these functional elements using experimental and computational methods [18]. The RESCUE-ESE method identified 238 ESEs that are preferentially associated with constitutive exons with weak splice sites [19]. In another study, octamers that are overrepresented in internal non-coding exons compared with pseudo-exons and 5' untranslated regions of intronless genes were compiled [20]. Putative ESSs were also identified by using *in vivo *[21] and computational methods [20,22]. These lists of putative elements provide a rich resource to study their functions in exon splicing.

To evaluate the contribution of alternative splicing to the evolution of new gene structures, we studied the evolution of splicing-related elements after gene duplication and determined their influence on splicing. The results can also help improve the understanding of functional divergence. We began with ESEs and ESSs, which are the two types of regulatory elements that are currently the best annotated. Using the synonymous substitution rate (Ks) between duplicates as a proxy for time after gene duplication, we find that ESEs and ESSs diverge with evolutionary time and the patterns are different between real and control motifs, indicating the effect of splicing. Furthermore, we observed that there is a tendency for duplicates to loose ESEs over time. Moreover, gains and losses of ESEs and ESSs tend to be concerted processes with one exon in a duplicated pair tending to always either gain or lose ESE or ESS. The net losses and gains are mainly caused by synonymous changes, which suggests that splicing selection is the major force influencing the splicing element changes. Consistent with the roles of ESE and ESS in splicing, we found that alternative exons have higher ESS and lower ESE densities when compared to their constitutive copies. And the fractions of exon pairs with different constitutive exons increase with duplication age. We conclude that ESE and ESS changes after gene duplication play important roles in alternative splicing divergence and contribute to the origin of new gene structures.

## Results

### ESE and ESS divergences increase with evolutionary time after gene duplication

**Figure 1:**
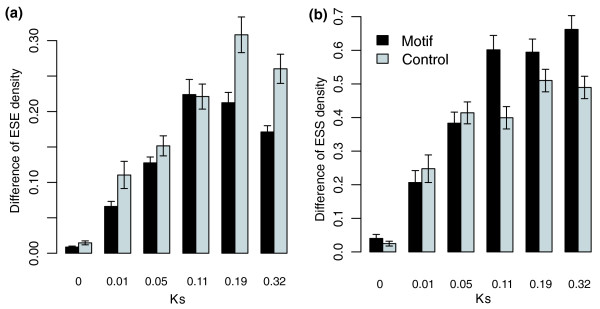
Both ESEs and ESSs diverge with the age of duplication. When compared to control motifs, it seems that divergence between paralogs is selected against in the case of ESEs and for in the case of ESSs by splicing constraints at the late stage. Error bars show standard errors in each group.

The relative difference of ESE and ESS density between two paralogous exons increases as Ks becomes large (Figure 1a, b). The spearman correlation coefficients for ESEs and ESSs with Ks are 0.536333 and 0.499158, respectively (Table 1). A detailed examination of the two graphs indicates that the difference grows faster in the early stage (Ks <0.11) and then reaches a plateau at the late stage. This pattern is quite similar to the evolution of protein sequences in chimeric fusion genes driven by adaptive evolution [23], suggesting that ESEs and ESSs possibly also undergo adaptive evolution after gene duplication.

**Table 1:** Spearman rank correlation of ESE and ESS divergence with Ks

	Paralogs (n = 2,074)		Orthologs (n = 99,716)	
		
	Rho	*P*	Rho	*P*
ESEs	0.536333	<2.2e-16	0.091868	<2.2e-16
ESSs	0.499158	<2.2e-16	0.170465	<2.2e-16

The numbers of pairs used are indicated in parentheses.

The observed large difference between paralogous exons implied the possibility of asymmetric evolution for ESEs and ESSs. To address this issue vigorously, we employ a statistical method to detect the difference between two paralogs based on the binomial distribution (see [24] and Materials and methods for details). If the two paralogous exons evolved symmetrically, the number of ESE or ESS elements specific to only one exon should be a random variable with a binomial distribution. As shown in Table 2, among the 1,089 pairs of paralogous exons, 166 show significant ESE difference (*P *< 0.05). However, since it involves 1,089 simultaneous statistical tests, about 1,089 × 0.05 ≈ 54 cases will show significance by pure chance at a significance level of 0.05, assuming the test is unbiased. Therefore, 166 - 54 = 112 cases are likely to be truly significant, which accounts for 10.3% of tested pairs. Similarly, for ESSs we observed 46 of 454 tested pairs of paralogous exons with *P *< 0.05, and 22 cases possibly happen by pure chance. Thus, 24 (5.1%) are truly significant. The proportion of exon pairs with asymmetric evolution of ESEs or ESSs is similar to the test of splicing isoform numbers (9.5%) [25].

**Table 2:** Asymmetric evolution of paralogous exons for ESEs and ESSs

	Total	Count (*P *< 0.05)
ESEs	1,089	166
ESSs	454	46

Shown here are the paralog pairs in which the total element changes are larger than 5 (see Materials and methods for details). The number of pairs with *P *< 0.05 is also given.

In most cases, ESE and ESS motifs in exons contain both protein-coding and splicing-related information. Therefore, evolution of these motifs should be constrained at two levels of selection - the protein sequence constraint and the requirement for accurate splicing - so called dual-coding [26]. To separate the two types of selection and see how splicing requirement affects ESE/ESS evolution, we constructed a set of control motifs using the method described by Ke *et al*. [27]. Briefly, we reversed and complemented each sequence of the original ESE set and then purged this set of sequences from the original set to obtain the control ESE set. The same procedure was applied to the ESS set. The control ESE and ESS sets have the same coherence among motifs and the same CG dinucleotide content as the original experimental sets. Compared with real motifs, these control motifs also code proteins, but do not presumably have splicing signals. Therefore, a comparison of the real ESE/ESS motifs to these control motifs can separate the effect of selection from splicing constraint. As shown in Figure 1a, real ESE divergence is generally lower than the control in all stages, and more so at the late stage. For the ESSs, the changes in the real motifs are slightly lower than in the control at the early stage, similar to ESEs, but the divergence exceeds that of the control set in the late stage (Figure 1b). These results suggest that splicing selection indeed has an effect on ESE and ESS changes after gene duplication, but the effects are different for ESEs and ESSs. For ESEs, selection for splicing may suppress their divergence to some extent, but for ESSs, the divergence is enlarged by selection on splicing at the late stage. This indicates that ESEs and ESSs have different evolutionary routes. This idea is consistent with another observation that ESEs are favored by exons but that ESSs are selected against during evolution [27].

One possibility to consider is that ESE/ESS divergence is a common phenomenon when sequence divergence occurs and is not actually related to the gene duplication event, for example, in a manner similar to changes between orthologs. To test this, we reanalyzed the relationships in human-mouse orthologous exons. Indeed, we found positive correlations between ESE and ESS differences and Ks (Table 1; rho = 0.091868 and 0.170465 for ESEs and ESSs, respectively). However, the magnitudes of correlations are much smaller than those that are observed between paralogs (Table 1). This result indicates that sequence divergence itself could also result in ESE/ESS divergence, but the degree is much stronger in paralogs.

### Gain and loss of ESEs and ESSs after gene duplication

The above result shows that both ESEs and ESSs diverge in the time after duplication. A more informative analysis would involve the comparison of the paralogs with their pre-duplication ancestors, from which creations or losses could be determined. The Inparanoid program is able to distinguish duplication events that happened before and after speciation and identify only the paralogs that were produced after speciation [28]. We used human and mouse proteins as inputs to identify paralogs in humans. Under these conditions, it is suitable to use mouse orthologs as the pre-duplication ancestors.

**Figure 2:**
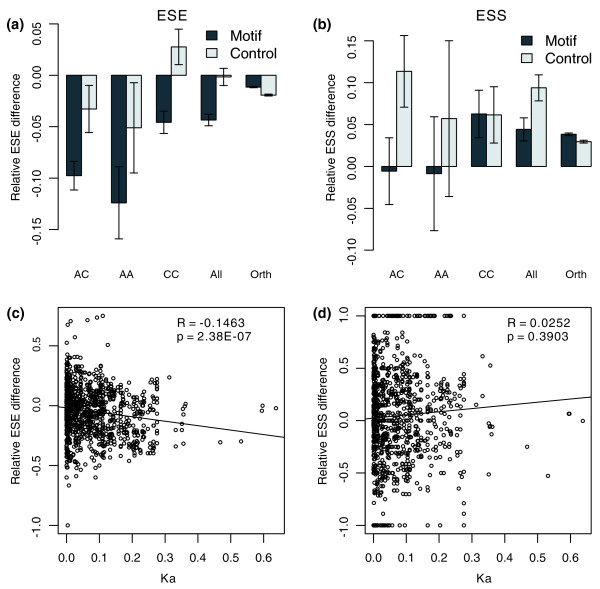
ESE and ESS gains and losses after gene duplication. **(a) **Overall losses of ESEs are observed in different types of paralogous exon pairs (AC, AA, CC) when compared to the genome-wide difference (Orth group). Futhermore, the AC and AA groups are significantly different from the CC group. Changes of control motifs are also plotted to see how splicing affects the evolution of ESEs. **(b) **Unlike the ESEs, no significant differences were found when compared to genomic differences (the Orth group). Similarly, the control motifs are displayed beside the ESS motifs. **(c) **The relative difference of ESEs is negatively correlated with Ka, and **(d) **no correlation is found for ESSs. A corresponding plot with Ks as time proxy is shown in Additional data file 2. Error bars in (a, c) show standard errors in each group.

The paralogous exon pairs, with their respective splicing information, were divided into three groups according to their splicing states, which were the AC, AA, and CC groups. Each exon pair in the AC group represents a pair of paralogous exons with one alternative and one constitutive exon, the AA group represents a pair of alternative exons, and the CC group represents two constitutive exons. The numbers for each group are listed in Table S1 in Additional data file 1. As a control to estimate the genomic bias, the differences between human and mouse orthologous constitutive exons without paralogs are displayed (this group is identified as Orth). We compared the mean value of ESE or ESS densities in each paralogous exon to the mouse ortholog to derive the element changes. The AC, AA and CC exon groups all show more ESE losses than the control (Orth group; Wilcoxon rank sum test, *P *= 5.97E-10, 0.0006909 and 0.0009766, respectively; Figure 2a). Furthermore, the AC and AA groups lost more than the CC group (*P *= 0.0015 and 0.01917, respectively). When all the paralogous exon pairs are included, the result is still significant (All versus Orth, *P *= 1.17E-07). For the ESSs (Figure 2b), the AC and AA groups have, on average, slightly more losses than the Orth group, but the differences are not statistically significant (Wilcoxon rank sum test, *P *= 0.9233 and 0.778 for AC and AA groups, respectively). The CC group and the 'All' group show similar changes to the Orth group. Interestingly, the ESE and ESS differences between orthologs (Orth group) do not equal zero. This emphasizes the necessity of using the orthologous exon pairs as a control.

We can also employ the shuffled motifs (as used in Figure 1), to see whether splicing requirements affect ESEs and ESSs when protein constraints are controlled. As shown in Figure 2a and Table S2 in Additional data file 1, generally the real ESE elements are lost more than the control ones for paralogous exons. In particular, compared to mouse exons, the average changes of control ESE motifs is around zero when all the paralogous exons ('All' group) are used, but a significant loss for real ESE motifs exists (two-sided Wilcoxon rank sum test, *P *= 0.002135). By contrast to the paralogous exons, the human-mouse orthologous exon pairs (Orth group) show greater conservation for the real ESE motifs than for the control (two-sided Wilcoxon rank sum test, *P *= 4.66 × 10^-15^). Different from ESEs, the changes of ESS elements in paralogous exons are smaller than the control ones, showing fewer gains of ESSs, especially when all the paralogous exons are used (*P *= 0.01321). Once again we observed a converse pattern in orthologous exons, showing more gains of ESSs than the control (*P *= 1.31 × 10^-8^). These observations suggest that splicing greatly affects the gains and losses of splicing elements in duplicates, and the pattern is different from that in non-duplicated genes.

It is also important to examine the ESE and ESS changes with the age of duplication. We use the Ka between duplicates as a proxy of duplication age. Compared to mouse, the ESE changes are negatively correlated with Ka (Spearman rank correlation analysis, rho = -0.1463, *P *= 2.38E-07; Figure 2c), which suggests that more ESE losses occur as the age of the duplication increases. For ESSs, as expected, there is no detectable correlation between the changes and Ka (rho = 0.0252, *P *= 0.3903) when the mean values of paralogous exon pairs are used (Figure 2d). We also use Ks as the proxy of duplication age and the same result is observed (Additional data file 2).

**Figure 3:**
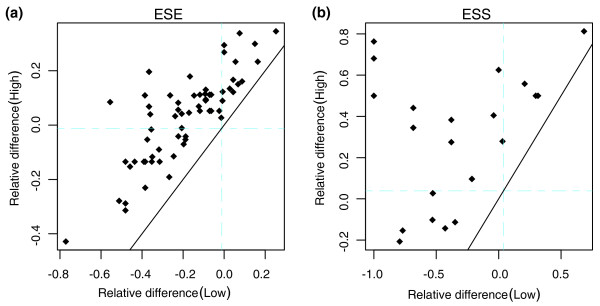
The diverse evolutionary pattern of ESEs and ESSs in asymmetric pairs of paralogous exons. The values of relative difference compared to mouse orthologs are plotted for each pair of paralogous exons. For easy visualization, we plot the copy with lower element density (Low) on the abscissa axis and the other with higher density (High) on the ordinate axis. The mean genomic difference for human-mouse orthologous exons are shown by the blue lines in each plot on both axes. The black diagonal line shows the pattern of symmetric evolution. **(a) **ESEs; **(b) **ESSs.

The above analysis is based on all the exons in each group of exons together. To explore further the underlying changes of paralogous exons, we examine the exon pairs showing statistically asymmetric evolution (Table 2). For easy visualization, we plot the member with lower ESE or ESS density in each pair of paralogous exons on the abscissa axis and the other with higher density on the ordinate axis (Figure 3); for easy description, we call the two copies 'Low' and 'High', respectively. As shown in Figure 3a, a significant portion of the exon pairs shows discordant changes, with one copy gaining ESEs and the other losing some, or with one copy changing and the other largely constant. We also found some pairs of exons showing gains or losses in both copies (the points near the black diagonal in Figure 3a), though the changing magnitudes are different. A similar pattern was observed for ESS changes (Figure 3b), but the points are sparse due to the small dataset. These observations display a diverse pattern of ESE and ESS evolution after gene duplication, and may reflect different requirements of functional divergence in different genes.

### Loss and gain of ESEs/ESSs are mainly caused by synonymous changes

Losses and gains of ESEs and ESSs in different copies of paralogous exons have been observed. Especially for the ESEs, only overall losses are found. It has been reported that protein sequences after gene duplications may diverge quickly [29,30]. Protein sequence changes are likely to disrupt and create ESEs or ESSs. Thus, it is possible that the changes in ESEs and ESSs are just a passive process that is caused by protein divergence rather than the requirement of splicing. A simple way to test this is to see which kind of mutations, synonymous or nonsynonymous, are more likely to result in ESE or ESS net gains and net losses. In other words, the synonymous changes do not affect the protein sequences and should be mainly affected by splicing selection.

**Figure 4:**
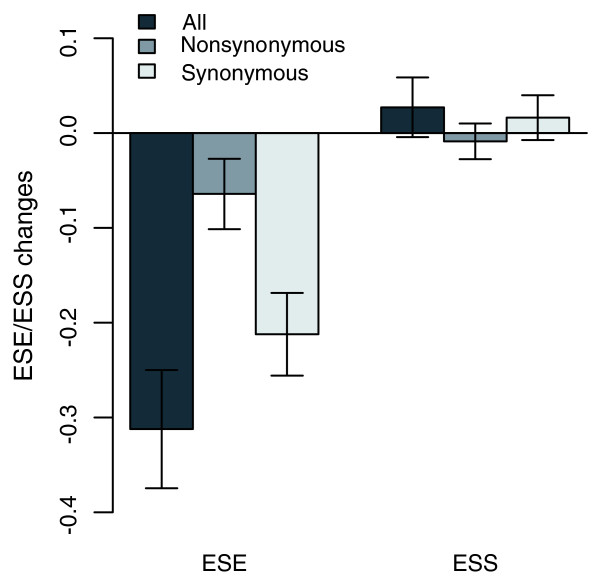
The creation and disruption of ESEs/ESSs in human duplicates. The ordinate axis denotes the average number of ESEs or ESSs that were changed in each exon. A negative value indicates a net disruption, and a positive value indicates a net creation. Significant disruptions of ESEs are found in both 'All' mutations and just synonymous mutations (see Table 3 for statistical tests). No significant net changes are found for the ESSs. Error bars show standard errors in each group. The data shown here merge the changes that occurred in each pair of paralogous exons. Please see Table 3 for the changes when the High and Low groups are considered separately.

To determine how disruption or creation of ESEs and ESSs happened after gene duplication, we used mouse orthologous exons as pre-duplication ancestors for comparison with the human paralogous exons in order to determine whether creation or disruption takes place. To exclude mutations that happened after the human-mouse split but before gene duplication, we only consider the mutational bases that are different between paralogous exons and either of which is the same as the mouse ortholog (see Materials and methods for details). Disruptions were subtracted from creations to derive the net changes, and any resulting positive values indicate net creations and negative values indicate net disruptions. When we calculate changes by combining the changes that occurred in both copies of each paralogous exon pair, which corresponds to the case above when the mean values of paralogous exons were used to determine gains or losses, the mean value of net ESE changes per exon pair when compared to the mouse orthologs is -0.312296 (Figure 4 and Table 3), which suggests more disruptions than creations (*P *= 1.54E-05) and is consistent with the above observation of more losses of ESEs (Figure 2a). As expected, the disruptions and creations for ESSs were equal, on average (mean = -0.0272, *P *= 0.3491; Figure 4 and Table 3). To discriminate the splicing selection from the protein sequence constraints, the mutations were further divided into synonymous and nonsynonymous groups. On average, each exon pair loses 0.2122 ESEs through synonymous changes, while this is 0.0642 for nonsynonymous changes (Figure 4 and Table 3). Furthermore, the changes that were caused by synonymous changes was significantly different from zero (*P *= 2.93E-06) but not for nonsynonymous changes (*P *= 0.1403). Again, no net ESS creations or disruptions that were caused by either synonymous or nonsynonymous changes were found. The same result was obtained when the changes were collected together (Table S3 in Additional data file 1).

**Table 3:** ESE and ESS net changes in human duplicates

		All	*P*	Non- synonymous	*P*	Synonymous	*P*
ESEs	Mean	-0.3123	1.54E-05	-0.0642	0.1403	-0.2122*	2.93E-06
	High	0.0228	0.4164	0.0308	0.2124	-0.0240	0.4879
	Low	-0.3147	7.6E-13	-0.0809	0.0023	-0.1870*	1.25E-08
							
ESSs	Mean	0.0272	0.3491	-0.0087	0.9629	0.0163	0.5235
	High	0.1106	1.37E-07	0.0285	0.0301	0.0604*	0.0001
	Low	-0.0992	1.72E-05	-0.0354	0.0095	-0.0559	0.0007

*The contribution of synonymous mutations to the net change of elements is significantly larger than that of nonsynonymous mutations (*P *< 0.05). In each cell, the number is the mean net change for each exon that is caused by mutations. Positive values mean more creation than disruption, and negative values the reverse. The 'mean' group merges the changes in each paralogous exon pair, while the High and Low groups include one of two paralogous exons with the higher or lower ESE/ESS density, respectively. The number of homologous clusters used in this analysis is 921.

The above analysis, by averaging the changes in different copies and clusters of paralogs, may obscure the underlying changes to some extent because the changes in different copies and in different paralogous exon pairs are discordant (Figure 3a, b). To support the above analysis further, each time we only examined the changes generated in the High or Low copy of each paralogous exon pair, as defined in the previous section. This method may enlarge the changes to some extent, but it is safe to test whether the changes are mainly caused by synonymous mutations. As shown in Table 3, in the High group, there were no net creations or disruptions for ESEs on average (mean = 0.022805, *P *= 0.4164), but there were significant net creations for ESSs (mean = 0.110604, *P *= 1.37E-07). Both synonymous and nonsynonymous mutations produced more ESS creations (*P *= 0.000106 and 0.03008, respectively), and the magnitude of net changes that were created by synonymous mutations was larger (Wilcoxon signed-rank test, *P *= 0.03114). In the Low group, net disruptions were observed for both ESEs (mean = -0.31471, *P *= 7.6E-13) and ESSs (mean = -0.0992, *P *= 1.72E-05). Moreover, the net changes produced by synonymous mutations are again larger (synonymous versus nonsynonymous, *P *= 0.01216 and 0.1173 for ESEs and ESSs, respectively). These findings are consistent with a previous report that the ratio of ESE/ESS changes are mostly caused by synonymous mutations [31] and suggest splicing itself is the major factor influencing ESE and ESS evolution.

### ESE and ESS changes influence exon splicing isoforms

ESEs and ESSs function as regulatory elements to activate or repress splicing of a certain exon [10,32]. The changes in these elements should contribute to the formation of different splicing isoforms (constitutive or alternative). To test this, we compared the changes of ESEs and ESSs between alternative and constitutive paralogous exons.

**Figure 5:**
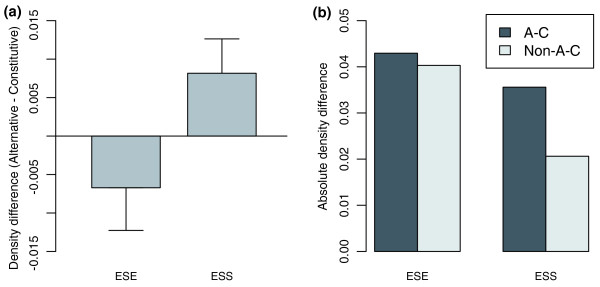
ESE and ESS divergence after gene duplication is associated with splicing state divergence. **(a, b) **Alternatively spliced exons show lower ESE and higher ESS densities than their constitutive copies (a) and absolute density differences between paralogous exons in AC (one alternative and one constitutive exon) pairs are larger than those in non-AC pairs for both ESEs and ESSs (b). Error bars in (a) show standard errors.

For comparison, all the paralogous exon pairs were divided into two groups: exon pairs with the same exon splicing state (AA or CC, non-AC group); and pairs with different types of exon splicing state (AC, AC group). The density of ESEs or ESSs between alternative and constitutive paralogous exon copies in AC pairs was compared in a pairwise manner. The alternative paralogous exon had significantly lower ESE and higher ESS densities than their constitutive copy (Wilcoxon signed-rank test, *P *= 0.02231 and 0.04243 for ESEs and ESSs, respectively; Figure 5a; Table S4 in Additional data file 1). The exon pairs with the same splicing states could not be deterministically divided into two groups, so the test for these pairs is unavailable. However, the absolute difference of ESE/ESS densities between paralogous exon pairs in AC pairs can be compared to those in non-AC pairs. If the ESE/ESS elements are functional, we expect that there should be larger differences in the AC pairs than non-AC pairs, and we do find a larger absolute difference in AC exon pairs for both ESEs and ESSs (Wilcoxon rank test, *P *= 0.04857 and 0.006637, respectively; Figure 5b). These results suggest that ESEs and ESSs are functional and that divergences in them can contribute to changes in exon splicing state. Usually, both ESEs and ESSs changed in one exon. There is currently no effective way to combine these two types of information so to do this we borrowed one parameter, the proportion of ESEs in an exon (ESE.prop), as defined by the number of ESEs divided by the sum of ESEs and ESSs in this exon [33] (see Materials and methods for details). This parameter is a relatively good predictor for splicing state, when other splicing signals are constant [33]. A larger ESE proportion means a higher probability for this exon to be included in spliced transcripts. Consistent with this property, alternative exons have a significantly lower ESE proportion than their constitutive partners (Table S4 in Additional data file 1). There was no statistical difference between alternative and constitutive exon copies with regard to the 5' and 3' splice sites (Table S4 in Additional data file 1). Unfortunately, there is not enough splicing information for mouse exons to determine the path of the splicing state changes of the human paralogous exons. Otherwise, we could examine more how ESE and ESS changes affect the splicing state shift.

Consistent with the above result, exons in the ESE Low group tend to be alternative when compared to the High group exons (28.70% versus 22.90%, *P *= 0.01642) (Table 4). In contrast, fewer alternative exons were observed in the ESS Low group (24.35% versus 27.25%), though this is not statistically significant (Table 4).

**Table 4:** Alternative and constitutive exon counts in the ESE/ESS High and Low groups

	High			Low			
			
	A	C	Proportion of A	A	C	Proportion of A	P*
ESEs	158	532	22.90%	198	492	28.70%	0.01642
ESSs	188	502	27.25%	168	522	24.35%	0.2424

A and C denote the alternative and constitutive exons, respectively. 'High' and 'Low' groups represent the member exons with higher or lower ESE/ESS densities in each pair, respectively. *P-value is determined from a test if the proportion of alternative exons is different between the High and Low groups.

**Figure 6:**
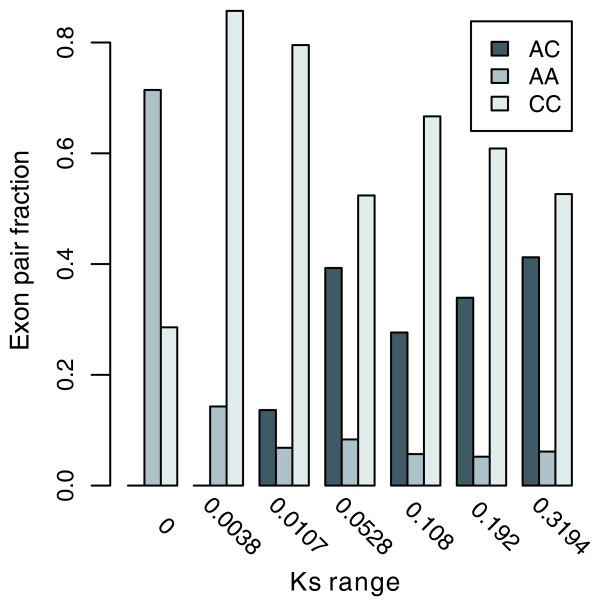
The fractions of different types of exon pairs with age of duplication.

ESE and ESS divergence increases with the age of the duplication (Figure 1). It may also be possible that more splicing state shifts will occur with evolutionary time. As predicted, the proportion of AC pair exons increases with duplication age (Spearman's rho = 0.8829187, *P *= 0.00845; Figure 6). Interestingly, a decrease of AA pair exons was also observed, although it was not significant (rho = -0.820032, *P *= 0.3879). However, the changes before Ks <0.011 are not reliable, since the exons with splicing information are too few to draw a reliable result (Additional data file 3).

## Discussion

Several mechanisms for the creation of new gene structures are known (reviewed in [4]). For example, duplication of *ymp *followed by recombination of retroposed *Adh *generated the *jingwei *gene [34]. However, evolution works like a tinker [35], and gene duplication may be only the first step. Subsequent changes to the duplicated gene are also important for sub-functionalization or neo-functionalization and determine the fates of duplicates [1,19,36]. In this study, we examined the divergence of splicing properties after gene duplication.

### ESE and ESS divergence contributes to new gene structures and functional divergence

It has reported that alternative exons could be derived from constitutive exons [31,37,38], possibly by weakening of 5' splice sites [39]. Exon splicing regulatory elements also play important roles in splicing state transitions [40], especially under weak splice sites [31]. Consistent with this idea, we observed lower ESE densities and higher ESS densities in alternative exons than in their constitutive copies (Figure 5a). We also checked if the alternative exons had lower splice site scores, but there was no difference (Table S4 in Additional data file 1). This result suggests that ESE/ESS changes, more than splice site changes, are responsible for splicing state transitions after gene duplication. Based on this, we speculated that one copy of the CC exon pairs with large ESE/ESS changes may be on the verge of becoming alternatively spliced, as any changes that relax the splicing signals would result in alternatively spliced exons. Consistent with our idea, a previous study proposed a model of alternative splicing evolution relevant to duplicated genes. The model depicts that alternative splicing isoforms are lost rapidly after gene duplication at an early stage and then new splicing isoforms evolve at a late stage (see Figure 2 in [25]). Our results support this model well (Figure 6). These new splicing isoforms that evolved after gene duplication are valuable sources of new gene structures. Some originally constitutive exons may be alternatively spliced in a minor mode and even lost in the future, depending on natural selection and genetic drift [31]. Similarly, some originally alternatively spliced exons may become constitutive when they are beneficial. Compared to other mechanisms of new gene structure origination, modifications of gene structures after gene duplication may require fewer changes and changes of otherwise low impact, thus rendering such a path to new structures relatively evolvable.

Divergence of splicing structures may also produce functional divergence among duplicates. It is easy to think that ESE/ESS changes that result in splicing state transitions will also result in new splicing isoforms, which will, in turn, acquire novel functions or partition ancestral functions. This may be realized mainly by two categories of changes. Alternative splicing could change functions by altering the expression of the gene, such as expression level or the place and time of expression. Alternative splicing is a subnetwork of gene regulation [16], and, thus, the changes could influence gene expression [32,41-44]. For example, alternative splicing is coupled with nonsense-mediated mRNA decay to regulate gene expression [45-47]. Previous studies showed that expression divergence is partially caused by changes in promoter regulatory elements and related *trans*-acting factors [48-50]. However, these changes could only explain 2 to 20% of expression divergence [48,49], and a weak relationship is also found in orthologs [51], which suggests that the promoter region is a weak predictor of gene expression levels. We hypothesize that alternative splicing is another possible way to alter expression. Since ESEs and ESSs are generally bound by specific factors in order to function, such as SR-proteins (for ESEs) and heterogeneous nuclear ribonucleoproteins (for ESSs) [10]. These factors may be expressed in a tissue-specific manner, such as nova [52-54]. These factors, combined with ESE/ESS divergence, could result in different expression among different tissues.

Consistent with this idea, Makova and Li [55] found that many duplicates are expressed differentially in different tissues. On the other hand, alternative splicing involves either inclusion or skipping of certain regions or exons in the gene, such that different protein products and structures are generated [56-59]. Alternative splicing could also change the reading frame in certain regions of exons and, in turn, encode different peptides [60]. Both mechanisms should be very important for the evolution of new functions or subfunctions that are different from the ancestral gene. Compared to coding sequence divergence, changes that are required in alternative splicing are maybe much more minor in order to reach the same degree of divergence [36]. However, it is possible that ESEs and ESSs may result in functional divergence in an alternative-splicing-independent manner. For example, ESEs may function as a transcriptional pause site [16], influence the transcription rate, and, in turn, produce different expression levels.

A previous study reported the negative correlation between coexpression and Ka [55]. In our study, we also found that both ESE changes are negatively correlated with Ka (Figure 2). This suggests that ESE/ESS changes are coupled with protein sequence changes. This is very important as protein sequence changes may help improve functionality when the protein is expressed in a different place or time. Furthermore, alternative splicing itself could alter the evolutionary rate of alternative exons or regions by lowering purifying selection pressure [61].

### ESEs and ESSs are selected at alternative splicing and protein levels

As ESEs and ESSs are mostly located within protein-coding sequences, their changes possibly affect both the protein sequence and splicing efficiency. Previous studies have proposed that ESEs constrained protein sequence changes and codon usage, especially in proximity to the exon boundary [27,62,63]. These studies suggest selection of splicing on ESEs inhibits the protein sequence changes and codon choices. On the other hand, protein sequence changes should also affect ESE/ESS motifs. Protein sequence divergence after gene duplication is important to evolve new functions [23,30]. Thus, the disruption and creation of ESEs/ESSs caused by protein sequences may be inevitable. Consistent with this idea, both creation and disruption could be caused by nonsynonymous changes (Table 3). However, these nonsynonymous changes may be slightly deleterious and the primary force may actually be from the splicing selections. In fact, the changes in ESEs/ESSs at nonsynonymous sites may be selected at two levels, protein and splicing [26]. It will be interesting to discriminate between the two types of selections and determine which is more predominant in future studies. As the two types of selections may not always coordinate, splicing selection should better operate at different places, such as synonymous sites, which are independent of protein sequence. Consistent with this, most ESE and ESS changes are caused by synonymous mutations (Figure 4 and Table 3). This result is also consistent with previous findings that synonymous sites show a much clearer trend toward increased evolutionary rate with increased distance from the splice sites than nonsynonymous sites [64], which suggests that splicing mainly acts on synonymous sites.

### The limitation of our studies

In this study, we do not include the intronic elements, such as intronic splicing silencer and enhancers, due to lack of validated global sets of these motifs. It will be interesting to determine how intronic elements have evolved after gene duplication and their relationships with ESEs/ESSs, and 5' and 3' start sites when the data sets are available. In fact, a significant challenge in this area is determining the method by which this information can be combined and by which exon splicing could be predicted [13,16,18,32]. This would heavily rely on our knowledge of splicing regulation. Although we found higher ESS and lower ESE densities in alternative exons than their constitutive copies, this is just an average trend and does not mean that exons with higher ESS or lower ESE densities are deterministically alternatively spliced. Changes in ESEs or ESSs alone could not predict exon splicing state accurately [65]. It is likely that understanding of splicing element changes will improve when the 'splicing code' [65,66] is well defined in the future.

We used the duplicates that occurred after the human-mouse split, about 91 million years ago [67]. Therefore, the duplicates in our datasets are relatively young. It will be interesting to see how ESEs/ESSs evolve when duplicates over a long evolutionary period are used in the future. On the other hand, since only a limited fraction of exons in our mouse set was mapped to the ASAP2 database [68], there is not enough exon splicing state information for the mouse exons, which prevents us from determining the direction of splicing state changes. This should be extended in future analyses, when mouse exon splicing data have accumulated. The newly emerging high-throughput sequencing technology has provided a powerful tool in this direction [47,69-72].

## Conclusions

Our results revealed the evolution pattern of exonic splicing elements after gene duplication and its influence on exon splicing. Furthermore the findings suggest that splicing requirement but not protein sequence mostly determines the changes of ESE and ESS. Combined with previous studies, our results imply that alternative splicing changes after gene duplication can have important roles in generating new splicing isoforms and gene structures.

## Materials and methods

### Identification of homologous exons

We downloaded the sequences and annotations for human and mouse from the NCBI RefSeq [73] database (build 36.1) [74] in January 2007. Any mRNAs for which the length in nucleotides of the coding sequence was not a multiple of three or that contain in-frame stop codons were excluded. The exon-intron structure information from the annotation files was then parsed using a Perl script.

To identify orthologs and paralogs for human and mouse, all the human and mouse proteins were input into the Inparanoid program (version 1.35) [28,75,76], which was run with the default parameters except for the overlap cutoff, which was set to 0.8 to improve specificity. The Inparanoid program is able to identify orthologs and in-paralogs (duplication that occurred after speciation) between two species. Therefore, we could obtain the orthologs between human and mouse and the paralogs in human, which were generated after human-mouse split (see the InParanoid FAQ [77] for details). Thus, it is reasonable to assume the mouse orthologs are the pre-duplication ancestors for the human paralogs.

To identify the homologous exons, the proteins in each paralog cluster were aligned using MUSCLE [78] and then converted to a coding sequence alignment using the BioPerl utility aa_to_dna_aln [79,80]. The exon-intron information was mapped on these alignments using Perl scripts. The paralogous exons were defined when they satisfied the following two criteria: the two exons must align at least at one boundary in the coding sequence alignment; and the two exons have the same length. We obtained 2,074 paralogous exons with this method (Additional data file 4). The same approach was applied to the orthologous genes between human and mouse, and 99,716 pairs of orthologous exons were identified (Additional data file 5). To improve the accuracy of homologous exon identifications, only mRNAs with the 'NM_' prefix, which generally have more experimental support, were used. The first and last coding exons in each mRNA were also excluded, as these exons are generally partially coding and may have different properties.

### Calculation of Ka and Ks

To calculate Ka and Ks, the coding sequence alignments generated above were input into the PAML package [81], and the values were calculated using the CodeML program with the parameter CodonFreq equal to F3X4, which is estimated from the nucleotide composition at each of three codon positions.

### Scanning of ESEs and ESSs in exons

The 400 ESE and 217 ESS hexamers were collected from supplementary Table S3 of Ke *et al*. [27]. These motifs combined the hexamers from Burge and colleagues [19,21] and those enriched in octamers from Zhang and Chasin [20]. These ESE and ESS hexamers were scanned separately in each exon using a Perl script, and their occurrences were denoted by the fraction of positions that were covered by motifs, which are given as *density*(ESE) and *density*(ESS), respectively. To quantify the changes (loss and gain) of ESEs and ESSs when compared to the mouse orthologs, the following formula, which is analogous to the method used by Corvelo and Eyras [82], was used:

where *density*_*human *_and *density*_*mouse *_are the motif densities for human and mouse genes, respectively, and the function max gets the larger value of the two. A positive value indicates a gain of motifs and a negative value indicates a loss of motifs. The ESE proportion for each is calculated as ESE count divided by the sum of ESE and ESS counts, like that described by Zhang *et al*. [33].

In order to get the control ESE and ESS motifs, as described in [27], the ESE motifs were converted into their reverse and complementary sequences, and original ESE sequences were purged from the converted sequences. The end result was 327 real ESE and 327 control ESE motifs. The same approach was applied to ESSs and resulted in 153 real ESS and 153 control ESS motifs. The new sets of motifs were searched separately, and compared to the real and control motifs.

In this study, we use the list of ESEs and ESSs that was compiled for humans and applied this list in a search of the mouse genome, since there is no validated ESS list for the mouse. This should have a systematic influence on the scanning of ESEs/ESSs in the mouse genome but should not change our conclusions, since we used the orthologous exon pairs as a control.

### Testing the asymmetric evolution of paralogous exons

To determine the asymmetric divergence of the two paralogous exons, we employ a method similar to that in [24]. Given a sequence alignment of two paralogous exons, we can scan the sequence one base at a time and detect the ESE or ESS hexamers specific to either exon and those shared by both sequences at the same position of the alignment. Assuming that there are totally n1 and n2 ESE elements observed in two paralogous exons and b of them are observed at the same position of the alignment, we regard these b ESE elements as conserved. Then there are d1 = n1 - b and d2 = n2 - b ESE elements specific to one of the two exons and these difference should be caused by gains and/or losses after gene duplication. If the two exons evolve in a symmetric mode, d1 and d2 should comply with the binomial distribution B(d1 + d2, 0.5). The probability *P *of observing a disparity as large as or greater than the actual one between d1 and d2 by chance alone is calculated by summing over the tails of the binomial distribution:

and *P *= 1 for d1 = d2. To minimize the noise, we exclude the exon pairs with the total difference d1 + d2 less than 6, which is the minimum to get a *P*-value of 0.05 in the most asymmetric situation. This resulted in 1,089 and 454 testable exon pairs for ESEs and ESSs, respectively.

### Splicing score calculation

The tool MaxEntScan [83,84] was used to calculate the scores for the 5' and 3' splice sites. The 5' splice site includes three bases in the exon and six bases in the intron. The 3' splice site includes 20 bases in the intron and 3 bases in the exon.

### Exon splicing state classification

To determine the splicing state (alternative or constitutive) for each exon, as described in [85], we mapped the ASAP2 database [68] exons to our exon sets by their positions on the chromosomes. The exon splicing states were then extracted from the ASAP2 database, which, in turn, allowed us to obtain the splicing states for our exons.

### Creation and disruption of ESEs/ESSs

To determine the mutations that create or disrupt ESEs/ESSs after gene duplication in human paralogs, the human exon coding sequences were aligned with mouse orthologous exons using their peptide alignment as a guide. For each pair of paralogous exons, only the mutational positions that were different within this pair of exons were considered, in order to ensure that the examined mutations occurred after gene duplication. The bases were compared to mouse orthologs at each mutation position in order to decide the mutational direction. Only those cases in which one of a pair of paralogous exons is the same as the mouse ortholog are considered, because this will enable determination of the sequence in which the mutation happened.

After determining the mutational direction, the six hexamers that overlapped the changed bases in the paralog were compared to aligned six hexamers in the ancestor to determine a net loss or gain of ESEs or ESSs. If there are more ESEs or ESSs in the ancestor, this mutation is likely a disruption, and, if it is vice versa, then the mutation is likely a creation. Note the two hexamers under comparison must contain only one mutation to exclude the possibility that the motif loss or gain is caused by mutations other than the one we are focused on.

## Abbreviations

ESE: exonic splicing enhancer; ESS: exonic splicing silencer; Ka: nonsynonymous substitution rate; Ks: synonymous substitution rate.

## Authors' contributions

XK, ZZ and LZ conceived and designed the experiments. ZZ and LZ performed the experiments. ZZ analyzed the data. LZ, PW, YL and XC contributed reagents/materials/analysis tools. ZZ, LZ, XK and LH wrote the paper.

## Additional data files

The following additional data are available with the online version of this paper: a document providing supplementary materials, including supplementary Tables S1, S2, S3 and S4 (Additional data file 1); a figure showing the relationship between ESE/ESS changes and Ks (Additional data file 2); a figure providing the counts of different types of paralogous exon pairs in each Ks group (Additional data file 3); a dataset listing all the human paralogous exon pairs used in this study (Additional data file 4); the complete list of human-mouse orthologous exon pairs used in this study (Additional data file 5).

## Supplementary Material

Additional data file 1 Supplementary Tables S1, S2, S3 and S4. Click here for file

Additional data file 2 This figures is same as Figure 2c, d, except that Ks is used as the proxy of gene duplication age. Click here for file

Additional data file 3 Plot of number of paralogous exons in each Ks group. Click here for file

Additional data file 4 This file contains the complete list of 2,074 paralogous exon pairs and their genomic information used in this study. Click here for file

Additional data file 5 The complete list of 99,716 human-mouse orthologous exon pairs used in this study and their genomic information. Click here for file
