# Supplementary material for: Divergence of exonic splicing elements after gene duplication and the impact on gene structures
Source: Genome Biol. 2009 Nov 2;10(11):R120. doi: 10.1186/gb-2009-10-11-r120 (PMC3091315; doi:10.1186/gb-2009-10-11-r120)
Supplement: Additional data file 1 — Supplementary Tables S1, S2, S3 and S4. [file gb-2009-10-11-r120-S1.doc]

### Supplementary materials

### TABLES

**Table S1** Counts of different types of paralogous exon pairs

| Type | AC | AA | CC | All^*^ |
| --- | --- | --- | --- | --- |
| Counts | 220 | 68 | 402 | 2074 |

*: ‘All’ includes all the paralogous exon pairs regardless their splicing states are known or not.

**Table S2** Comparison of gain and loss between real and control ESE/ESS elements

|  |  |  | AC(159) | AA(36) | CC(299) | All(1236) | Orth(97130) |
| --- | --- | --- | --- | --- | --- | --- | --- |
| ESE | Real | Mean | -0.09765 | -0.12404 | -0.0458 | -0.0435 | -0.01147 |
|  |  | SE | 0.013866 | 0.034991 | 0.010825 | 0.005627 | 0.000611 |
|  | Control | Mean | -0.0328 | -0.0511 | 0.027557 | -0.00164 | -0.01925 |
|  |  | SE | 0.022882 | 0.043884 | 0.017254 | 0.008382 | 0.000907 |
|  |  | p | 0.01402 | 0.2239 | 0.008746 | 0.002135 | 4.66E-15 |
| ESS | Real | Mean | -0.0056 | -0.00862 | 0.062641 | 0.044245 | 0.038417 |
|  |  | SE | 0.039807 | 0.06795 | 0.028286 | 0.013772 | 0.001597 |
|  | Control | Mean | 0.113488 | 0.057087 | 0.061528 | 0.093839 | 0.029594 |
|  |  | SE | 0.042763 | 0.092995 | 0.0336 | 0.015528 | 0.001658 |
|  |  | p | 0.05328 | 0.7472 | 0.9462 | 0.01321 | 1.31E-08 |

Notes: Mean, the mean value of element changes compared to ancestral states in each group of exons; SE, the standard error of the mean; p, the two-sided Wilcoxon rank sum test was employed to test the difference between real and control motifs in each group. The numbers of exon pairs used are shown in the parenthesis following exon pair types.

**Table S3** ESE and ESS creations and disruptions in human duplicates

|  |  | Synonymous | |  |  |  | Non-  synonymous | |  |  |
| --- | --- | --- | --- | --- | --- | --- | --- | --- | --- | --- |
|  |  | Disruption | Creation | Ratio^c^ | p-value^b^ |  | Disruption | Creation | Ratio | p-value^b^ |
| ESE | Sites^a^ | 321 | 197 | 1.63^*^ | 5.66E-08 |  | 200 | 170 | 1.18 | 0.1315 |
|  | Motifs^a^ | 490 | 295 | 1.66^*^ | 3.42E-12 |  | 310 | 251 | 1.24 | 0.0143 |
| ESS | Sites^a^ | 98 | 110 | 0.89 | 0.4457 |  | 52 | 60 | 0.87 | 0.5085 |
|  | Motifs^a^ | 131 | 146 | 0.90 | 0.4003 |  | 78 | 70 | 1.11 | 0.5652 |

‘Ratio’ is the ratio of disruption to creation.

a: ‘Sites’ and ‘Motifs’ denote the changes in units of mutational sites and of motifs, respectively. For example, there are 321 synonymous mutations disrupting ESEs and, in all, 490 ESEs are disrupted by these mutations.

b: p-values are derived from tests that whether the number of disruptions equals to the number of creations using the binom.test in R package.

c: Chi-squared test is employed to compare whether the ratio in synonymous changes is different from that in nonsynonymous changes. Only the values labeled with ‘*’ are statistically different between synonymous and nonsynonymous groups.

**Table S4** The splicing signal density difference between alternative and constitutive copies in AC pairs of exons

|  | Mean^a^ | p |
| --- | --- | --- |
| ESE | -0.00671 | 0.02231 |
| ESS | 0.008163 | 0.04243 |
| ESE.prop | -0.0173 | 0.000395 |
| 5'ss score | 0.027407 | 0.8532 |
| 3'ss score | 0.013287 | 0.3956 |

a: the mean value of differences from all exon pairs
