# Supplementary figures and images for: Divergence of exonic splicing elements after gene duplication and the impact on gene structures
Source: Genome Biol. 2009 Nov 2;10(11):R120. doi: 10.1186/gb-2009-10-11-r120 (PMC3091315; doi:10.1186/gb-2009-10-11-r120)

**A**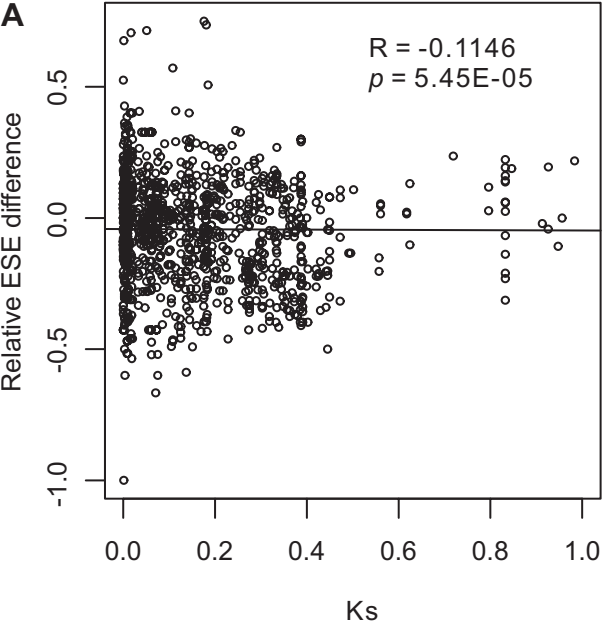**B**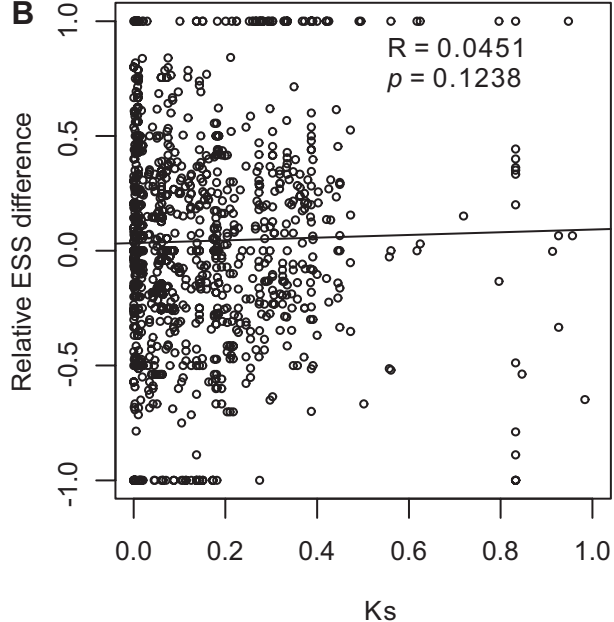

Supplement: Additional data file 2 — This figures is same as Figure 2c, d, except that Ks is used as the proxy of gene duplication age. [file gb-2009-10-11-r120-S2.pdf]

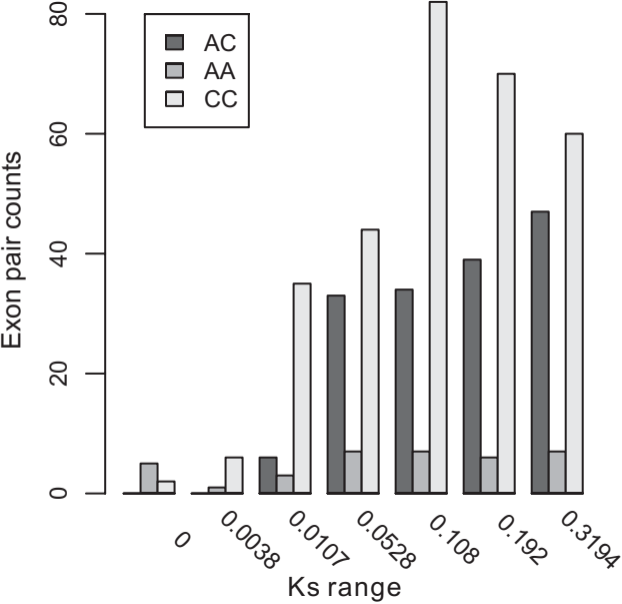

Supplement: Additional data file 3 — Plot of number of paralogous exons in each Ks group. [file gb-2009-10-11-r120-S3.pdf]
